# Large simple randomized controlled trials—from drugs to medical devices: lessons from recent experience

**DOI:** 10.1186/s13063-025-08724-x

**Published:** 2025-01-21

**Authors:** Sergio Buccheri, Stefan James, Marion Mafham, Martin Landray, Tom Melvin, Jonas Oldgren, Richard Bulbulia, Louise Bowman, Lotje Anna Hoogervorst, Perla J. Marang-van de Mheen, Peter Juni, Peter McCulloch, Alan G. Fraser

**Affiliations:** 1 Department of Medical Sciences, Cardiology and Uppsala Clinical Research Center, Uppsala University, Uppsala, Sweden; 2 Clinical Trial Service Unit and Epidemiological Studies Unit (CTSU), Nuffield Department of Population Health, University of Oxford, Oxford, UK; 3 School of Medicine, Trinity College Dublin, Dublin, Ireland; 4 Department of Orthopaedics, Leiden University Medical Center, Leiden, The Netherlands; 5 Safety & Security Science and Centre for Safety in Healthcare, Delft University of Technology, Delft, The Netherlands; 6 Nuffield Department of Surgical Sciences, University of Oxford, Oxford, UK; 7 Department of Cardiology, University Hospital of Wales, Cardiff, UK

**Keywords:** Randomized controlled trials, Large simple trials, Medical devices

## Abstract

Randomized controlled trials (RCTs) are the cornerstone of modern evidence-based medicine. They are considered essential to establish definitive evidence of efficacy and safety for new drugs, and whenever possible they should also be the preferred method for investigating new high-risk medical devices. Well-designed studies robustly inform clinical practice guidelines and decision-making, but administrative obstacles have made it increasingly difficult to conduct informative RCTs. The obstacles are compounded for RCTs of high-risk medical devices by extra costs related to the interventional procedure that is needed to implant the device, challenges with willingness to randomize patients throughout a trial, and difficulties in ensuring proper blinding even with sham procedures. One strategy that may help is to promote the wider use of simpler and more streamlined RCTs using data that are collected routinely during healthcare delivery. Recent large simple RCTs have successfully compared the performance of drugs and of high-risk medical devices, against alternative treatments; they enrolled many patients in a short time, limited costs, and improved efficiency, while also achieving major impact. From a task conducted within the CORE-MD project, we report from our combined experience of designing and conducting large pharmaceutical trials during the COVID-19 pandemic, and of planning and coordinating large registry-based RCTs of cardiovascular devices. We summarize the essential principles and utility of large simple RCTs, likely applicable to all interventions but especially in order to promote their wider adoption to evaluate new medical devices.

## Key points/Highlights

• Large simple RCTs of drugs and medical devices are feasible and may be simplified using modern IT infrastructure and technology.

• RCTs need to be adequately large in order to get reliable answers to their clinical questions.

• RCTs need to be efficient by minimizing additional work for patients and doctors in order to ensure that adequate numbers (and ideally, diversity) of patients are enrolled.

• RCTs need to focus information on important clinical outcomes.

• Use of clinical registries, platform, or other methods can help achieve these goals.

## Introduction

Randomized clinical trials (RCTs) provide the foundation of evidence-based medicine [1]. Randomly assigning participants to different therapeutic strategies is the best way to minimize sources of bias and allows inference of causality between interventions and their clinical outcomes [2, 3]. Well-designed and accurately conducted RCTs robustly inform clinical practice guidelines and decision-making processes, but barriers to their conduct include high costs related to excessive complexity in the governance of trials, and limited generalizability to patients receiving the intervention in daily clinical practice when only highly selected cohorts of patients are studied [4]. Large simple RCTs including a more representative patient population can address both problems.

The Medical Device Regulation (MDR) that came into effect in the European Union (EU) in May 2021 provides a regulatory framework that aims to balance the efficient approval of new medical devices (or technical iterations of existing devices) with demonstration of their safety. It requires evidence to be presented in a Clinical Evaluation Report (CER) that supports the intended use and safety of a medical device, before market approval, and then periodic reports on safety to be submitted thereafter [5]. The MDR imposes higher standards for generating and assessing evidence on the performance and safety of high-risk medical devices (class III and implantable devices) than was required under the previous medical device directives, and it specifies in particular that “clinical investigations shall be performed for implantable and other high-risk medical devices” [6]. However, more specific methodological aspects of clinical investigations (i.e., type and/or design of studies) are not addressed in detail in the MDR. This creates uncertainty about what is considered sufficient clinical evidence and which types of studies are appropriate—particularly for new high-risk devices, for which there is generally a dearth of information [7, 8].

The principles of simplifying the design and avoiding unnecessary distractions in the conduct of RCTs were developed many years ago [9]. They have been reconfirmed for “streamlined” RCTs of drugs [6, 7] and demonstrated to be feasible for conducting large and simple RCTs of high-risk medical devices [10, 11]. Nowadays, the increasing availability of routinely collected healthcare data (for example, in registries) and the continuing development of more powerful information and communication technologies provide new opportunities for applying these concepts much more widely.

The objectives of the Coordinating Research and Evidence for Medical Devices (CORE-MD) project, led by the European Society of Cardiology and the European Federation of National Associations of Orthopaedics and Traumatology, are to review methodologies of clinical investigations and to advise on optimal study designs for high-risk medical devices [12]. Importantly, members of the consortium have pioneered the design and conduct of large, simple RCTs both of drugs and of medical devices. Sharing knowledge accumulated through that experience may be useful to apprise others of their unique value and to foster their wider adoption when obtaining evidence for regulatory approval. The objectives of this viewpoint are to identify the basic principles and to summarize the most important features of large simple RCTs.

## Reducing obstacles to performing RCTs

Strict and inflexible (over-) interpretation of the International Council for Harmonization (ICH) Good Clinical Practice (GCP) Guidelines has placed ever-increasing demands on the conduct of RCTs [4]. Although ICH GCP recommendations are aimed primarily at drug trials, to acquire evidence for licensing, they have been considered relevant also for trials of medical devices. They were designed to safeguard patients while promoting the utility and transparency of RCTs, but now the bureaucratic burden imposed on institutions, clinicians, and research staff is perceived as overwhelming [13, 14]. General standards for RCTs of medical devices (such as ISO 14155:2020) do not differ substantially from ICH GCP recommendations.

Unnecessary and time-consuming hurdles can discourage patients from participating in trials. A lack of interest in reducing complexity, by actors with a vested interest in the conduct and oversight of RCTs such as Clinical Research Organizations, may also limit the design and conduct of new RCTs [15]. The GCP recommendations are being revised by ICH (for details see ICH E6 (R3) at https://www.ich.org/page/efficacy-guidelines), but it is unclear how much this new guidance will reduce bureaucratic obstacles.

Simplifying the conduct of RCTs, without reducing their quality, is of paramount importance to increase the number of RCTs being performed and to reduce their costs [4]. Generating more high-quality clinical evidence will be useful for regulators, to increase the confidence and accuracy of their decisions to approve new drugs or medical devices. It will also benefit patients by upholding their right to receive treatments that are effective and safe. Essential principles have been summarized by the Good Clinical Trials Collaborative (GCTC, link at https://www.goodtrials.org/) (see Table 1) and are applicable to trials of devices as well as other interventions. They stress the importance of avoiding unnecessary distractions during the conduct of RCTs, such as excessive monitoring of data that are not of key relevance, unnecessarily complex procedures for reporting clinical information and adverse events, and the need for investigators to accomplish redundant administrative processes [16].

**Table 1:** Examples of principles, implications, and recommendations from the Good Clinical Trials Collaborative

Principle	Implications	Recommendations regarding:
Relevance and utility	Design characteristics of RCTs should be aimed to resolve important uncertainties about the effects of a health intervention	- Appropriate population - Robust intervention allocation - Adequate size - Blinding and masking of interventions - Adherence to allocated interventions - Completeness of follow-up - Relevant measures of outcomes - Proportionate, efficient, and reliable capture of data - Ascertainment of outcomes - Statistical analysis - Assessing beneficial and harmful effects of the intervention - Monitoring emerging information on benefits and harms
Respect of participants	Ethical responsibilities regarding participants, future and current patients, and the public	- Appropriate communication - Relevant consent - Changing consent - Implications of changing consent - Managing the safety of individual participants - Communication of new information relevant to the intervention
Collaboration and transparency	Practices that contribute to develop trust between all those involved in an RCT and generalize confidence in the RCT ecosystem	- Working in partnership with people and communities - Collaboration among organizations - Transparency
Appropriateness for their context	Ensuring that a trial is set up to be practicable and produce reliable, actionable results	- Setting and context - Use of existing resources
Efficiency and management	Competent decision-making and coordinated execution based on good governance and good trial quality management	- Competent advice and decision-making - Protecting trial integrity - Planning for success and focusing on issues that matter - Monitoring, auditing, and inspection of study quality

## Landmark large simple RCTs of drugs and interventions

**Fig. 1:**
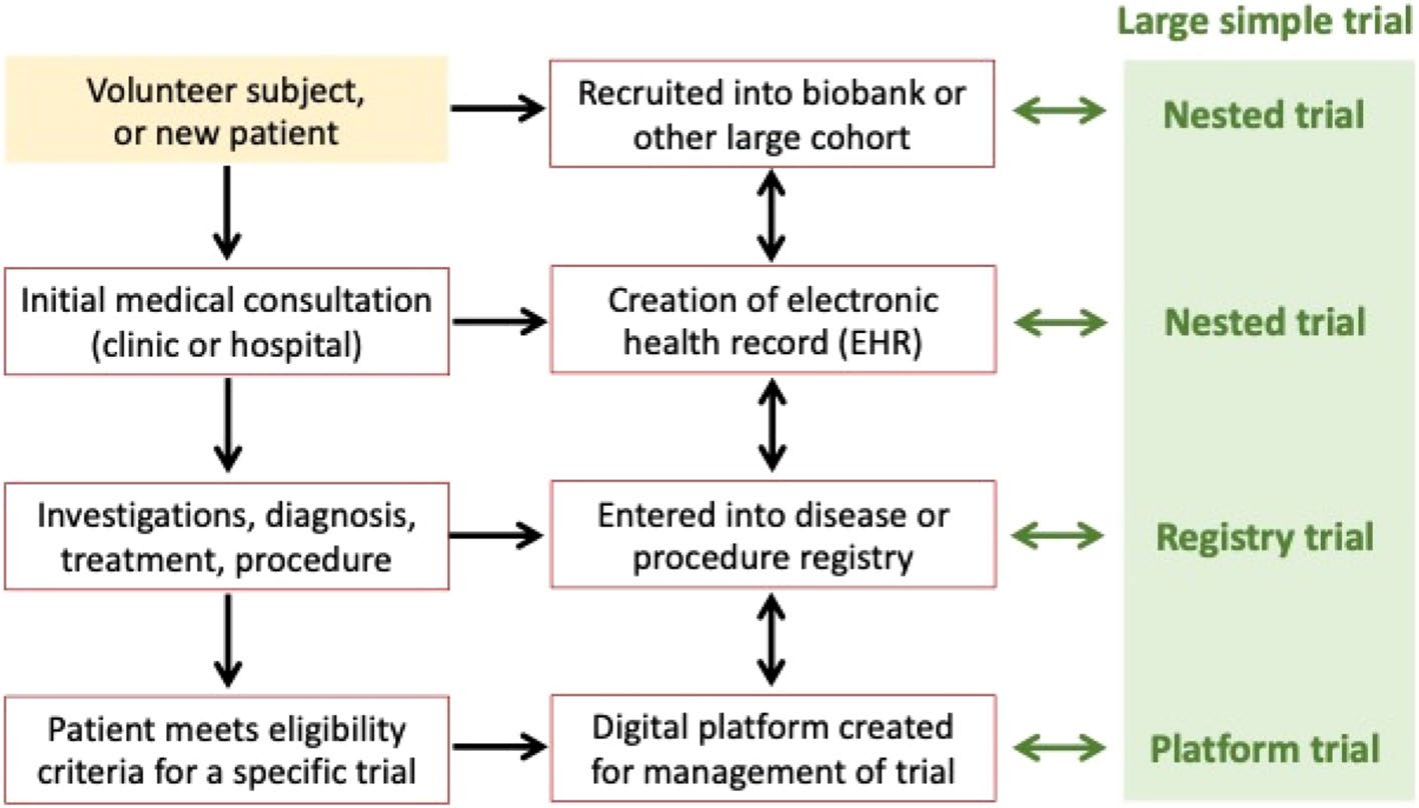
Variants of large simple trials. Whether the subject is a volunteer or a patient, and however they qualify for a clinical study or trial, and regardless of the electronic record or computerized database used as the framework for a large simple clinical trial, the essential principles remain the same. The left-hand column shows the common sequential stages of initial assessment and investigation of a patient; the middle column illustrates the types of electronic databases into which data from the subject or patient may be entered at each stage; and the right-hand column lists terminologies commonly applied to trials using those databases. Collectively, these are described as “large, simple trials”

Many different terms have been proposed to describe study designs and methodologies that share the key features of randomization, simplicity in study conduct (leading to large sample sizes), and efficient management and data collection (achieved by exploiting existing electronic platforms and databases, see Fig. 1). The single umbrella term “large simple trial” covers all these options, including platform trials (such as RECOVERY), registry trials (such as TASTE), and nested trials. The conceptual foundation of a large simple RCT is to make and keep its design and conduct as streamlined as possible. It should be inclusive and affordable, and able to provide results that are widely generalizable to real-life clinical practice [17–19].

RCTs can be simplified by establishing easier processes for collecting information, using short case report forms and linking with data that are acquired routinely as part of the delivery of healthcare (including national databases, claims data, and disease-specific registries) [20–27]. The feasibility of this approach has been demonstrated in large RCTs of drugs and medical devices [11, 28]; two key examples are the RECOVERY trial, evaluating drugs for COVID-19, mainly conducted in the UK [29], and the TASTE trial, evaluating a medical device for patients with acute myocardial infarction, mainly conducted in Sweden [10, 28].

### The RECOVERY trial

During the COVID-19 pandemic, there was an urgent need for reliable evidence about pharmacological interventions to treat the effects of the SARS-CoV-2 virus, but little capacity within front-line hospitals to deliver research, so the “Randomised Evaluation of COVID-19 Therapy” (RECOVERY) trial was initiated in March 2020 as a platform trial. As of December 2024, it continues to assess the effects of potential therapies on all-cause mortality in patients hospitalized with COVID-19. The trial was conceived as a large simple trial drawing heavily on the example of the second International Studies of Infarct Survival (ISIS-2) conducted in the 1980s [30]. A key factor in the success of ISIS-2 was the recognition that by keeping the workload associated with enrolling participants into the study to an absolute minimum, it was possible to embed the trial in the everyday work of busy hospitals where most heart attack patients are treated.

Using a similar approach, all aspects of the RECOVERY trial were streamlined by design (see Table 2 for an overview of its key design features) [31]. Simple eligibility criteria include hospitalization with proven or suspected COVID-19, with the local investigator being allowed to assess suitability for each of the trial treatments according to local guidelines. The trial was open-label to enable rapid implementation, and it used a combination of parallel-group, sequential and factorial randomizations to assess potential therapies in an adaptive design. The primary outcome was all-cause mortality at 28 days, with secondary outcomes including the duration of hospital stay, and a composite end-point of death or need for invasive mechanical ventilation or extracorporeal membrane oxygenation among patients not on invasive mechanical ventilation at baseline. During maximum recruitment, 185 hospital sites across the UK were taking part, and since February 2021 non-UK sites have been included across seven countries [32]. By April 2023, over 48,000 participants had been randomized to one or more comparison and the trial had already delivered 13 practice-changing results (see https://www.recoverytrial.net/).

**Table 2:** Features of large simple randomized trials compared with more conventional designs

Feature	Conventional design	RECOVERY	TASTE
Design	Sophisticated and controlled. Limited use of alternative strategies (such as factorial and/or adaptive designs)	Platform-based factorial design enabled multiple treatments to be assessed rapidly	Use of on an ongoing registry (SCAAR) for allowing a streamlined conduct of the study
Consent	Long and complex consent form, excessive training requirements for site staff	Short 3-page information leaflet, 20-min self-directed training for site staff, a doctor independent of the study team could serve as the legal representative for patients unable to provide consent	Information provided by the treating physician at the time of primary percutaneous coronary intervention. Verbal consent accepted in the acute phase before the intervention. Simplified informed consent provided to the patient
Eligibility criteria	Complex criteria requiring laboratory or other results and extensive exclusion criteria	Simple criteria that can be determined easily by the treating clinician	Simple criteria that can be determined easily by the treating clinician using the information collected routinely in the registry
Baseline assessments	Complex assessments including collection of biological samples, clinical measurements, or disease severity scales	Minimal data collection by site staff (e.g., demographic characteristics, ventilation status, other COVID-19 therapies, and major co-morbidities) supplemented by linkage to healthcare systems data	No extra activities for collecting baseline information. All information already collected in the registry
Outcome data collection	Long follow-up eCRF, detailed data collection, adjudication of outcomes	Minimal data collection by site staff supplemented by linkage to healthcare systems data, no outcome adjudication	No extra activities for outcome collection. All events obtained using national registries
Monitoring	Excessive source data verification	24-h telephone support for site staff, central monitoring of recruitment and randomization balance, independent ascertainment of study outcomes by linkage to healthcare systems data, independent Data Monitoring Committee to make recommendations based on unblinded analyses of safety and efficacy data	No monitoring and/or outcome adjudication
Long-term follow-up	Rarely possible	Low-cost long-term follow-up through linkage with healthcare systems data	Low-cost long-term follow-up through linkage with healthcare systems data

*Abbreviations*: *eCRF* electronic case report form, *COVID-19* coronavirus disease 2019

Data collection by local site staff was minimal. A one-page electronic case report form (eCRF) is completed at randomization, and again at the earliest of 28 days later, hospital discharge, or death. In the UK, data collected by local sites are supplemented from National Health Service (NHS) datasets and national registries, using the NHS number which uniquely identifies each participant. The linkage of RECOVERY participants to more than 40 national datasets (predominantly coded data collected for health service planning and reimbursement and National Registries) aimed to:

1. Ensure complete follow-up information for the main trial outcomes, even when participants are transferred for care between hospitals
2. Provide additional baseline characteristics (e.g., ethnicity), reducing on-site data collection
3. Enable long-term follow-up of participants beyond 28 days
4. Avoid the need for source data verification, by providing an independent source of information for the primary outcome
5. Allow assessment of additional outcomes not captured by the follow-up eCRF

### The TASTE trial

The “Swedish Web-system for Enhancement and Development of Evidence-based care in Heart disease Evaluated According to Recommended Therapies” (SWEDEHEART) was launched in 2009 and collects data consecutively on all patients with different cardiac conditions (such as acute or chronic coronary syndromes, heart valve disease, or cardiac rehabilitation) who require specialist medical management or interventional or surgical therapies [33].

Patients are informed about their proposed inclusion in SWEDEHEART when they present to a cardiology service, and they are registered using their personal identification number (PIN), a unique 12-digit number that each Swedish inhabitant receives at birth or on immigration into Sweden, mainly for taxation purposes. Written and verbal information is given but no specific informed consent is requested at the time of initial registration in SWEDEHEART, and patients can at any time-point deny consent for registration as well as opt out during follow-up. All information collected by caregivers is transferred directly to a central server located at the Uppsala Clinical Research Center. SWEDEHEART is connected to the Swedish National Population Registry for obtaining continuous information on vital status. The PIN may, after signed informed consent, be used to collect specific follow-up data by merging study data retrieved from SWEDEHEART with other national health care registries (e.g., hospitalization, cause of death, and drug prescription registries).

The limitations of using observational data for inferring causality have generated concerns and skepticism about the reliability of (adjusted) observational findings using data collected in a registry [34, 35]. For example, in an exceptional case related to the early evidence of outcomes from first-generation drug-eluting coronary stents (DES), adjusted observational findings from the registry suggested an increased risk of death at 1 year with DES versus bare metal stents. Excessive reactions to this early report impacted routine clinical practice [36]. In Sweden, the clinical use of DES (rather than bare metal stents) dropped significantly, from about 60% in 2005 to 15% in 2007 [36]. Initial concerns about an increased risk of death with DES were not confirmed by long-term results from RCTs. In other examples, early evidence from observational data has been confirmed by subsequent trial results. Well-conducted observational research that has minimized bias by its design [37] and implemented appropriate statistical approaches [38] may give more accurate results than a poorly conducted and underpowered RCT—but a large and well conducted RCT is preferable when possible.

The feasibility and value of using the web infrastructure of SWEDEHEART to overcome the limitations of observational data, by randomizing patients to different treatment strategies or interventions, was demonstrated in the “Thrombus Aspiration in ST-Elevation Myocardial Infarction in Scandinavia” (TASTE) trial, which was the first (medical device) registry-based randomized clinical trial or “R-RCT” [28, 39].

TASTE compared the routine manual aspiration of intracoronary thrombus before percutaneous coronary intervention (PCI) versus standard PCI without thrombus aspiration in patients with acute myocardial infarction undergoing primary PCI [40]. Thus, it investigated high-risk medical devices (all CE-marked manual aspiration catheters) used as part of a therapeutic strategy. The design of TASTE was kept very simple [40] by employing a minimal set of exclusion criteria and by obtaining the primary endpoint of all-cause mortality by direct linkage with the Swedish Population Registry. A minimal administrative burden was imposed on investigators by using clinical and follow-up information that was already collected in the SWEDEHEART registry and by avoiding separate monitoring and adjudication of adverse events (see Table 2).

Pre-procedural data were registered as patients entered the PCI lab. The system helped investigators to check inclusion and exclusion criteria and then randomized eligible patients within a few seconds. In this acute clinical setting, obtaining a verbal informed consent and randomizing patients directly at the time of the procedure was a necessary pre-requisite for the trial to be conducted successfully. All patients were asked to confirm their agreement to participate by providing written informed consent within 24 h.

**Fig. 2:**
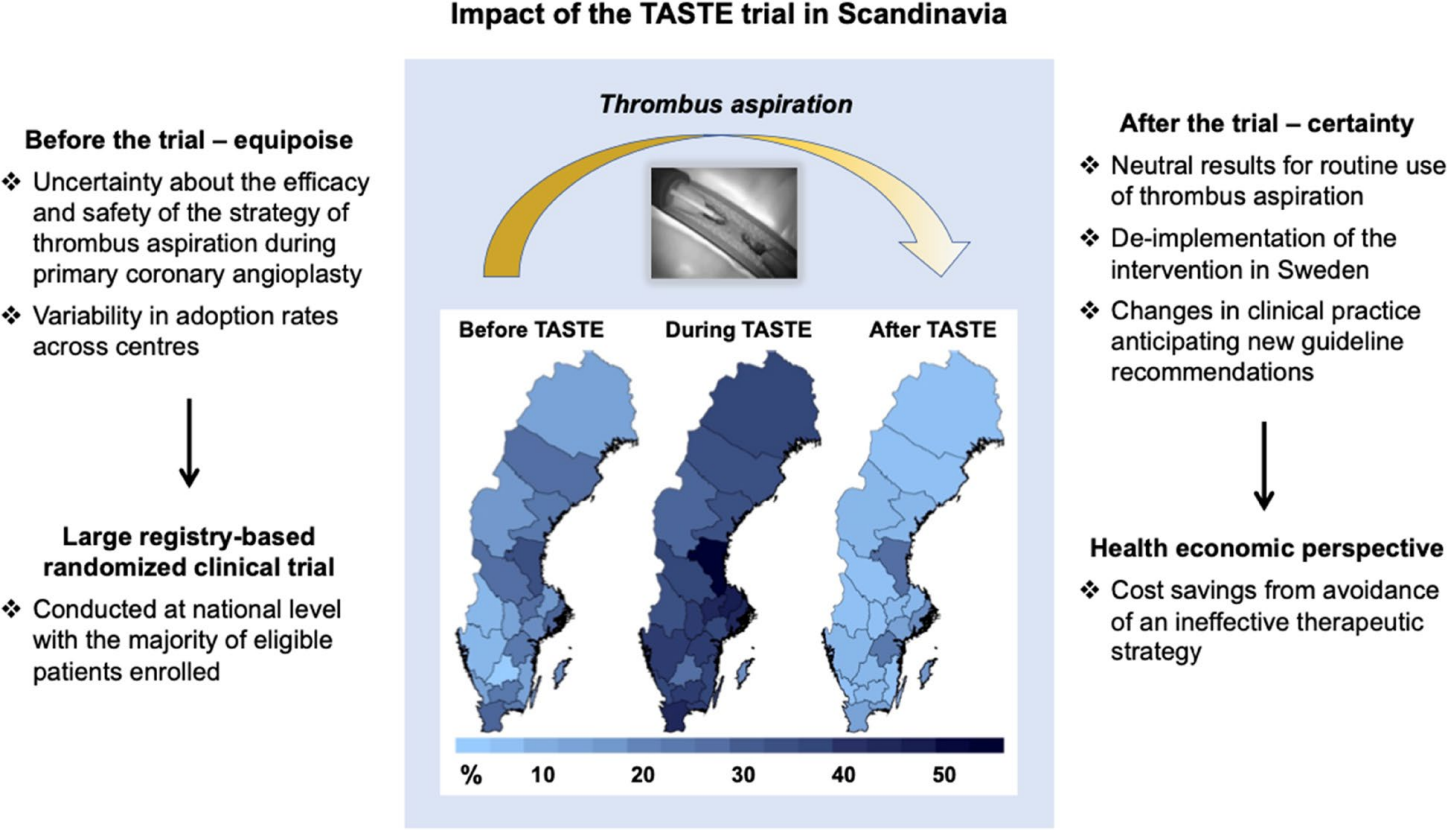
Impact of the first registry-based randomized trial of a medical device. Illustration of the major impact that was achieved by the first registry-based randomized controlled trial (the TASTE study) [10]. In TASTE, 82% of all potentially eligible patients (in Sweden and Iceland) were enrolled [25]. This figure shows the percentages of all consecutive patients who had ST-elevation myocardial infarction (i.e., not only those enrolled in the trial) who received thrombus aspiration, in different Swedish regions before, during, and after the TASTE trial

All hospitals performing PCI in Sweden, with the addition of one center in Denmark and one in Iceland, contributed to the screening and randomization of 7244 patients within less than 3 years. Routine thrombus aspiration had no impact on mortality at 30 days or at 1 year [28, 41], so the findings led to substantial de-implementation of thrombus aspiration in Sweden (Fig. 2), even before a class III recommendation for its routine use during primary PCI was issued in European guidelines [10].

## Principles of large simple RCTs

What lessons can be learned from the RECOVERY and TASTE trials? The paradigm of using RCTs to assess the causal effect of an intervention on outcomes, and of using registries only later for post-market clinical follow-up, has now substantially shifted [34]. RCTs of drugs in acute emergency settings such as the ISIS-2 and RECOVERY trials have similarities with trials of medical devices such as TASTE. Firstly, they are most likely to be successful in recruiting large numbers of participants if they are fully embedded in usual clinical care pathways, for which a streamlined approach to all aspects of trial design is essential. Secondly, in contrast to long-term drug trials in chronic conditions, they are less reliant on long-term engagement with participants themselves. Instead, high levels of adherence require effective engagement with healthcare professionals within the care pathway, and long-term capture of the occurrence (or, more challenging, the lack of occurrence) of relevant outcomes—which makes such trials suitable for remote, decentralized re-use of healthcare systems data.

### “Large and simple” versus “pragmatic” RCTs

The scope and definition of large simple RCTs overlap with those of “pragmatic” RCTs, potentially making the additional term redundant. However, while the term “pragmatic” is now widely used, it specifically refers to the expectation that pragmatic RCTs closely mimic routine clinical practice (e.g., delivery mechanisms and RCT settings) in most or all key elements [42]. Large simple RCTs are generally, though not necessarily, pragmatic in nature, as certain elements of their design may diverge from routine practice. The term “large” is crucial, as this characteristic is not inherently emphasized in the definition of a “pragmatic RCT.” Being sufficiently and appropriately large is essential for RCTs to overcome random errors and achieve greater precision in estimating treatment effects for important outcomes.

### Conduct of large simple trials

Data collected as part of routine healthcare delivery have been successfully re-used in numerous observational studies but have not been utilized as extensively in RCTs. Barriers to the use of healthcare systems data for collecting outcomes in RCTs include:

- Failure to collect sufficient consent to cover data linkage activities
- Lack of expertise within clinical trial teams for data engineering
- Challenges related to information governance (including reluctance to release participants’ identifiers to the coordinating center or sponsor, to allow linkage with national datasets)
- Concerns from funders and healthcare regulators about the completeness and accuracy of outcomes ascertainment
- Lack of national healthcare datasets collated by organizations with mechanisms to undertake data linkage

Existing or possible cross-border collaborations (such as the European Health Data & Evidence Network and the European Health Data Space) should ensure that linkage of healthcare data with national and international trial cohorts is prioritized.

Rather than any specific novel aspect of their simplified study conduct, the efficiency, quality, and chances of final success of any large simple RCT are enhanced by applying general principles that guide the design and conduct of all RCTs (https://www.goodtrials.org/guidance). These include randomized allocation to an intervention without foreknowledge of the assigned treatment, adherence to the randomized intervention, complete follow-up, and unbiased collection and analysis of outcome data.

Large simple RCTs attempt to minimize unduly restrictive exclusion criteria, which simplifies and speeds up enrolment [43]. Larger cohorts of patients provide more precise estimates of the treatment effect of an intervention (its internal validity). Broad inclusion criteria may help to ensure that the risk profile of patients included in large simple RCTs will be similar to that expected in routine practice (providing external validity) [44]. Ideally, all late-phase clinical trials and certainly all large simple trials should be generalizable to standard clinical practice. In comparison, RCTs that have been conducted only in well-defined and restricted cohorts of patients may lack sufficient power to provide compelling evidence on important clinical outcomes. There have been many prominent instances when the results of observational studies and smaller RCTs have deviated substantially from the findings of large RCTs (see Table 3).

**Table 3:** Examples of divergent outcomes observed in non-randomized and randomized cardiovascular studies

Type of device	Observational study	Smaller RCT	Larger RCT
First-generation drug-eluting stents versus bare metal stents	PMID: 17,296,822 Year: 2007	PMID: 12,050,336 Year: 2002	PMID: 14,724,301 Year: 2004
	- Propensity-score adjusted analysis (*n* = 19,771) - Higher risk of death with DES versus BMS	- 1:1 randomization (*n* = 238) - No in-stent restenosis with DES - No episodes of stent thrombosis - No differences in mortality	- 1:1 randomization (*n* = 1314) - Marked reduction in restenosis and repeat revascularization with DES - No differences in mortality
ABSORB bioresorbable vascular scaffold versus everolimus-eluting metallic stent	PMID: 26,875,648 Year: 2016	PMID: 27,806,897 (ABSORB II) Year: 2016	PMID: 26,457,558 (ABSORB III) PMID: 30,266,412 (ABSORB IV) PMID: 31,553,222 (ABSORB III-LTFU) PMID: 37,207,924 (ABSORB IV-LTFU) Year: 2015 to 2023
	- Propensity-score matched (*n* = 905 paired matches) - No differences in clinical outcomes	- 2:1 randomization (*n* = 501) - No difference in vasoreactivity, and higher late luminal loss, with ABSORB - Higher rate of TV-MI with ABSORB	ABSORB III: - 2:1 randomization (*n* = 2008) - Noninferiority of BVS versus EES for TLF met at 1 year - Higher rates of TLF, TV-MI, and scaffold thrombosis through 5 years ABSORB IV: - 1:1 randomization (*n* = 2604) - Noninferiority of BVS versus EES for TLF met at 30 days and 1 year - Higher rates of TLF through 5 years
Manual thrombus aspiration versus standard PCI	• PMID: 20,550,973 • Year: 2010	PMID: 18,256,391 (TAPAS) PMID: 18,539,223 (TAPAS-FU) Year: 2008	PMID: 23,991,656 (TASTE) PMID: 25,853,743 (TOTAL) PMID: 25,176,395 (TASTE-FU) PMID: 26,474,811 (TOTAL-FU) Year: 2013 to 2016
	- Multivariable adjustment (*n* = 22,632) - Increased risk of death with thrombus aspiration (RR, 1.16, 95% CI 1.05 to 1.28)	- 1:1 randomization (*n* = 1071) - Better reperfusion and clinical outcomes with thrombus aspiration - Reduced risk of cardiac death with thrombus aspiration	TASTE: - 1:1 randomization (*n* = 7244) - No differences in mortality at 30 days and 1 year TOTAL: - 1:1 randomization (*n* = 10,732) - No differences in the composite outcome of cardiovascular adverse events at 30 days and 1 year - Increased risk of stroke with thrombus aspiration
Intra-aortic balloon pump versus standard of care in patients with cardiogenic shock and AMI	PMID: 11,376,306 Year: 2001	PMID: 19,770,739 Year: 2010	PMID: 22,920,912 Year: 2012
	- Multivariable model in a large disease registry (*n* = 23,180) - IABP in combination with thrombolytic therapy associated with reduced mortality	IABP-SHOCK: - 1:1 randomization (*n* = 45) - No statistically significant effects on reduction of severity of disease, improvement of cardiac index, reduction of inflammatory state, or reduction of BNP biomarker	IABP-SHOCK II: - 1:1 randomization (*n* = 600) - No differences in mortality at 30 days and 1 year
Embolic protection device during TAVI	PMID: 32,972,578 Year: 2020	PMID: 27,815,101 Year: 2017	PMID: 36,121,045 Year: 2022
	- Propensity-score matched (*n* = 1575 paired matches) - Use of embolic protection devices associated with a lower incidence of ischemic stroke and in-hospital mortality	- 1:1:1 randomization (*n* = 363) - Embolic protection did not change neurocognitive function - No difference in new lesion volume on MRI	PROTECTED-TAVR: - 1:1 randomization (*n* = 3000) - No differences in stroke within 72 h after TAVR

*Abbreviations*: *RCT *randomized controlled trial, *PMID *PubMed identifier, *DES* drug-eluting stent, *BMS* bare metal stent, *TV-MI* target vessel myocardial infarction, *BVS* bioresorbable vascular scaffold, *EES* everolimus-eluting stent, *TLF* target lesion failure, *IABP *intra-aortic balloon pump, *MRI *magnetic resonance imaging, *TAVR* transcatheter aortic valve replacement

Small, focused RCTs can generate initial insights into the efficacy and safety of an intervention using surrogate markers to obtain results over shorter duration of follow-up, and they may help to refine a hypothesis and inform the design of a subsequent large RCT. Studies of medical devices during their early development should ensure that evidence is collected concerning the feasibility of the procedure, protocols for implantation and use, variability in operator practice, and operator learning curves. Then, the appropriate type of RCT depends mainly on the stage of development of the drug or medical device. Initially, it is advisable to assess the value of a new intervention in small-sized, highly controlled studies. If the safety and efficacy profile is promising, then larger confirmatory RCTs should be used to establish evidence for policy recommendations regarding its implementation. When conducted well, large simple RCTs have a greater potential to inform methods for improving public and population health, due to their robust external validity and generalizability. Large simple RCTs will be most efficient when the intervention is widely available and can be delivered to a large number of patients in a short time.

## Large simple RCTs of medical devices: feasibility and challenges

Experience has now confirmed that national registries can be used successfully as platforms for screening, randomization, and follow-up of patients treated with high-risk (class III) medical devices [28]. As demonstrated in TASTE, R-RCTs embedded within an ongoing device or disease registry are able to enroll large numbers of patients in a relatively short amount of time, so they will impact clinical practice [25, 39]. Although medical devices in lower-risk classes generally do not require RCTs for market approval, large and simple RCTs may be feasible for these categories, offering potential advantages such as reduced costs and faster, more comprehensive enrolment.

There may be situations where more detailed information about baseline characteristics and technical details of the index procedure can be important and relevant, and sometimes more information needs to be collected about adverse events during follow-up, to define a more granular composite primary endpoint. To be able to deal with such issues, technical refinements of the infrastructure supporting the conduct of R-RCTs have been made. In the case of SWEDEHEART, the registry provides a direct link to a computerized R-RCT framework which is a web application developed by the Uppsala Clinical Research Center. It provides a randomization module and a unique link between the patient’s registry file and the trial electronic data capture system (EDC). The EDC can thereby collect additional baseline, procedural and outcomes data from other sources or by direct data entry. Also, active monitoring and central adjudication of adverse events have been implemented in contemporary R-RCTs in Sweden. The R-RCT design concept developed for TASTE has been used and further developed in several large R-RCTs of drugs and medical devices, including a recent double-blind placebo-controlled R-RCT [45]. These iterations have not affected the conceptual framework of simplifying the conduct of large trials that remains central to the design of R-RCTs, but they have broadened the landscape of the types and nature of R-RCTs of medical devices that can be successfully conducted (see Table 4).

**Table 4:** Completed and ongoing R-RCTs of high-risk medical devices in Sweden

R-RCT name	Device investigated	Number of patients	Registry used for screening/randomization	Primary endpoint	Type of monitoring/adjudication	Funding for the trial	Status of completion
TASTE	Thrombus aspiration catheters	7244	SWEDEHEART–SCAAR	All-cause death	None	- Swedish Heart Lung Foundation - Terumo Medical and Medtronic	Completed
iFR-SWEDEHEART	Pressure wire and software for coronary functional assessment	2037	SWEDEHEART–SCAAR	Composite of all-cause death, non-fatal MI, and unplanned revascularization	Clinical event committee for non-fatal MI and unplanned revascularization	- Volcano Corporation	Completed
SWEDEPAD	Drug-eluting technology (stents, balloons) in PAD	~ 2500	Swedish Vascular Registry (SWEDVASC)	- Amputation rate in patients with critical limb ischemia - Health-related quality of life in patients with claudication	No adjudication DSMB in the trial	- Swedish Research Council, Swedish Heart–Lung Foundation, and Region Västra Götaland - All companies selling drug-coated balloons and drug-coated stents	Ongoing (interim analysis on mortality published)
HipSTHeR	Arthroplasty implants	1440	Swedish Fracture Registry	Composite all-cause death and re-operation	None	Swedish Research Council, Swedish Society of Medicine, Deltofs foundation, The Geriatric fund, Uppsala-Örebro Research Council, ALF funding	Ongoing
DUALITY	Dual mobility cups	1600	Swedish Fracture Registry	Any dislocation of the index joint treated with closed or open reduction within 1 year after surgery	None	Research grant from the Swedish Research Council	Ongoing
INFINITY	Drug-eluting stents	2400	SWEDEHEART–SCAAR	Composite of cardiovascular death, target vessel myocardial infarction, and ischemia-driven target lesion revascularization	Clinical event committee DSMB	Unrestricted research grant from Elixir Medical Corporation	Ongoing

*Abbreviations*: *MI *myocardial infarction, *DSMB* data safety monitoring board

### Challenges for large simple RCTs of medical devices

The quality of all RCTs depends on the extent to which bias is avoided in all phases [46], i.e., when allocating subjects to one of the investigational arms; when ascertaining, processing, and analyzing outcomes; and when ensuring adherence to the allocated intervention (minimizing cross-over), along with obtaining complete follow-up data [47]. In RCTs of medical devices, randomization just before a procedure may help to reduce any risk of cross-overs or non-adherence to the assigned treatment. Bias may arise if patients or investigators are aware of the randomized assignment [46], which can occur if there are major differences between arms in the nature or intensity of how outcomes are ascertained. This is much less likely with objectively assessed clinical outcomes than those that are more subjective, but even an objectively assessed outcome such as all-cause mortality can be biased if there are differences in completeness of follow-up between the intervention and control arms (i.e., attrition bias). Follow-up through linkage with healthcare systems data can help to ensure complete ascertainment of outcomes, independently of any affect which knowledge of the treatment allocation might have on participant engagement with the trial.

### Prevalence of the disease

As shown by recent examples, it is easier to design and conduct large simple RCTs for diseases that are common or moderately common. Diseases with rarer incidence pose more challenges if RCTs are needed to test novel medications and/or devices, requiring a high degree of internal control and making a simple design difficult to implement. Further challenges may include the need to enroll a large number of sites across many countries, with substantial heterogeneity of routinely collected healthcare data. Screening and recruitment can be facilitated if there is a specific disease registry, or a broader registry tracking rarer conditions. The BROKEN-SWEDEHEART trial, an R-RCT investigating different therapeutic pharmacological strategies in patients with takotsubo syndrome, will provide information about specific challenges and the likelihood of successful completion for large R-RCTs in rare pathological conditions [48].

### Diversity and inclusivity

Ensuring diversity and inclusivity are important objectives for large simple RCTs. Minimization of exclusion criteria, a key element in their streamlined design, is expected to result in the inclusion of a high proportion of eligible patients, including those who belong to previously underserved groups (who are represented in health research at lower levels than would be expected based on population estimates) [49]. Large simple RCTs are not immune to this risk, however. Concerns were raised about the under-representation of Black, Asian, and minority ethnic (BAME) subjects in the RECOVERY trial [50], although eventually they constituted one-sixth of patients included in the trial. In the VALIDATE trial, an R-RCT testing the use of bivalirudin versus heparin for anticoagulation of patients with acute myocardial infarction, differences were seen between the baseline characteristics of patients included in the trial, and those who had been screened and fulfilled inclusion criteria but were not eventually included [51].

Diversity and inclusivity are complex issues in RCTs. Regrettably, research on effective strategies to promote the inclusion of underserved groups remains limited [52]. Cultural and communicative barriers should be considered during the design and screening phases and addressed by, for example, providing informed consent materials in different languages or specifically training research staff to support underserved patients.

### Blinding using sham procedures

Double-blinding of both patients and investigators is the ideal approach to remove potential sources of bias arising from knowledge of the assigned treatment in RCTs [53]. It is common in pharmacological trials, but often problematic in RCTs of non-pharmacological interventions [54]. Blinding of patients in RCTs of medical devices can be ensured by performing a sham procedure that mimics the active intervention in all aspects including the route of surgical access, the duration of the procedure, and any post-procedural diagnostic assessments [55, 56], but for obvious reasons operators cannot be blinded. Sometimes information obtained by medical imaging or the nature of scars can reveal which type of device has been implanted. To minimize biases, operator roles should be limited in later RCT activities such as contacts with patients and the recording of outcomes. Examples of proper blinding using sham procedures in RCTs of cardiovascular interventions have been reported [57].

It is not easy to implement blinding via sham procedures, however, either in large simple RCTs of high-risk medical devices or for other surgical interventions. A sham procedure imposes extra costs and time, and it deviates from standard clinical practice. In head-to-head comparisons of different devices that are implanted using the same procedure (for example, comparing different drug-eluting stents during PCI), single-blinding of patients can be sufficient to reduce bias. Before the procedure and randomization, it should be stressed to the patient that he or she will not receive any information on the type of device that will be implanted, and afterwards blinding of patients can be maintained by training and instructing research staff and by avoiding any specific entry into the clinical records of the type of device that has been used. An example of successful implementation of effective procedures to ensure single-blinding in R-RCTs is the INFINITY-SWEDEHEART trial (NCT 04562805).

### Operator learning curves and selective inclusion of centers

The technical skills of surgeons and other operators can be improved and refined through performing more interventions [58, 59]. Learning curves for complex or new procedures are demonstrated when progressive improvements in efficacy and safety reach a plateau [60]. Ignoring the experience of individual operators during RCTs of medical devices may lead to inaccurate estimates of the outcomes of an intervention. Ideally, a device implanted via a complex procedure should be tested in an RCT once the technical proficiency of all operators in the study has reached the plateau phase. Investigations to understand and define learning curves should be encouraged, and virtual simulation of complex procedures may help in developing technical standards for operators who will participate in RCTs [61, 62].

Large simple RCTs of medical devices should therefore be conducted once their implantation techniques have matured and been standardized. Otherwise, starting a large RCT for a new and complex procedure could expose patients to unnecessary risks and could produce an unreliable assessment of the value of a new technology if compared to existing interventions with which the operators are familiar. The particular value and optimal role of large simple RCTs of medical devices, therefore, can be to investigate iterations of existing medical devices or to assess new devices that are delivered or implanted through established procedures (for example, comparing drug-eluting stents that are implanted using standard techniques).

In conventional industry-funded RCTs of medical devices, the intervention is generally delivered in a highly controlled setting in high-volume centers. Outcomes of a complex intervention using medical devices may be very different in such specialized centers as compared to routine clinical care. By expanding the number of centers participating in a study, large simple RCTs mitigate the risk of overinterpreting the (proportional) effect of an intervention before it is transferred to standard clinical practice.

### Willingness to randomize and be randomized

RCTs can be performed ethically when there is genuine uncertainty about the preferred treatment of a specific disease—namely a state of equipoise [63–65]—but strong beliefs among investigators and/or patients about the value of an unproven intervention (novelty bias) may lead to the selective and unrepresentative inclusion of patients, for example, from lower-risk categories [66]. Even worse, strong prior beliefs may make randomization impossible if no patients are screened for inclusion. This aspect is particularly important for RCTs of medical devices if there is eager anticipation about the value of an active intervention, leading to reluctance to enroll subjects or for patients to consent if the comparator arm involves no device implantation. For these reasons, it is crucial to share detailed information about the existing gaps in evidence that lie behind the need to conduct an RCT, with both investigators and eligible patients.

Where there is a perceived high risk of investigator bias, leading to a biased presentation of the evidence to patients, they should be protected from this by training investigators to present the facts in a neutral way [67] during the informed consent process, or by substituting them with trained nurses or computer decision-support programs [68].

### Costs and funding of trials

Trials have substantial costs [69] and performing conventional RCTs has become prohibitively expensive particularly due to resources needed to collect and monitor huge amounts of data, which sometimes are not even crucial for the primary outcome of the trial. Data collection using existing platforms offers potential advantages in terms of cost, efficiency, and completeness, and critically it is not dependent on action from participants or site staff and therefore it is relatively unaffected by knowledge of the treatment allocation in open-label studies. Cost reduction has been substantial in RECOVERY; based on a final expenditure of £20 million for the trial (plus the cost of the drugs), it has been calculated that the cost per patient/per answer was less than £40 (about €45).

Despite TASTE being relatively inexpensive (entailing mainly the standard costs of maintaining the registry), its findings were consistent with the results of a conventional and significantly more expensive industry-funded RCT investigating the same research question [70].

High costs are a particular disincentive for creating essential evidence for medical devices, since substantial investment is required for their development and testing as well as for accessing the market [71]. In Europe once a medical device has obtained the Conformité Européenne (CE) mark, there are limited incentives for manufacturers to raise the level of supporting evidence by demonstrating incremental benefit from a new device in a large pivotal RCT. Fear of negative results, alongside the need for more investment, can make it impossible or uninteresting for companies to strive for better clinical evidence.

The possibility of conducting large simple RCTs of high-risk medical devices should become less dependent on, but not uncoupled from, industry funding. Ideally, the infrastructure of registries required to evaluate medical devices should be paid for and maintained by public institutions or government, while research foundations and manufacturers should support individual trials. Regulatory incentives for conducting large RCTs would be crucial, and rigorous health economic assessments would be valuable. Demand from the medical community for reliable data from large RCTs could serve as a powerful incentive for conducting this type of studies.

In Sweden, many R-RCTs have been financed successfully by industry-independent research grants and public funding [28], while the costs of maintaining the national quality registries used in R-RCTs are met by the Swedish public health care providers and the government [33]. Economic support by industry partners has been also used in Swedish R-RCTs of medical devices, for example, by the free donation of devices and through institutional research grants.

## Conclusions

More efficient methods of generating reliable clinical data on the safety and performance of drugs and high-risk medical devices are necessary. Adequate clinical evidence is crucial for supporting regulatory decisions and for ensuring that market approval is awarded to medical devices that provide benefits to patients. Whenever possible, and according to the stage of development, the conduct of RCTs should be more strongly supported and in some cases required by regulatory guidance. Large simple RCTs can provide robust answers about the performance and safety of drugs and medical devices, so they should be encouraged whenever feasible but especially adopted more widely to evaluate new medical devices.

## Data Availability

Not applicable.
