# Correction: Large simple randomized controlled trials—from drugs to medical devices: lessons from recent experience

**DOI:** 10.1186/s13063-025-08783-0

**Published:** 2025-03-06

**Authors:** Sergio Buccheri, Stefan James, Marion Mafham, Martin Landray, Tom Melvin, Jonas Oldgren, Richard Bulbulia, Louise Bowman, Lotje Anna Hoogervorst, Perla J. Marang-van de Mheen, Peter Juni, Peter McCulloch, Alan G. Fraser

**Affiliations:** 1 Department of Medical Sciences, Cardiology and Uppsala Clinical Research Center, Uppsala University, Uppsala, Sweden; 2 Clinical Trial Service Unit and Epidemiological Studies Unit (CTSU), Nufeld Department of Population Health, University of Oxford, Oxford, UK; 3 School of Medicine, Trinity College Dublin, Dublin, Ireland; 4 Department of Orthopaedics, Leiden University Medi- Cal Center, Leiden, The Netherlands; 5 Safety & Security Science and Centre for Safety in Healthcare, Delft University of Technology, Delft, The Netherlands; 6 Nufeld Department of Surgical Sciences, University of Oxford, Oxford, UK; 7 Department of Cardiology, University Hospital of Wales, Cardif, UK

**Correction** **:** **Trials 26, 24 (2025)**

**https://doi.org/10.1186/s13063-025-08724-x**

Following the publication of the original article [1], we were notified that the name of the 10^th^ author was incorrectly tagged in the article’s XML.

**Incorrect tagging:**

 <GivenName>Perla</GivenName> 

 <GivenName>J.</GivenName> 

 <GivenName>Marang-van</GivenName> 

 <Particle>de</Particle> 

 <FamilyName>Mheen</FamilyName> 

**Correct tagging:**

 <GivenName>Perla</GivenName> 

 <GivenName>J.</GivenName> 

 <FamilyName>Marang-van de Mheen</FamilyName> 

The original article has now been corrected.

## References

[1] Buccheri, et al. Large simple randomized controlled trials—from drugs to medical devices: lessons from recent experience. Trials. 2025;26:24. 10.1186/s13063-025-08724-x. 39833917 10.1186/s13063-025-08724-x PMC11749104

